# Optimum Mix Design and Correlation Analysis of Pervious Concrete

**DOI:** 10.3390/ma18174129

**Published:** 2025-09-02

**Authors:** Fenting Lu, Li Yang, Yaqing Jiang

**Affiliations:** School of Civil Engineering, Wanjiang University of Technology, Ma’anshan 243031, China; wt16017@wjut.edu.cn (F.L.); wt14021@wjut.edu.cn (L.Y.)

**Keywords:** pervious concrete, correlation analysis, orthogonal test design, mix design

## Abstract

Pervious concrete is challenged by the inherent trade-off between permeability and mechanical strength. This study presents a systematic optimization of its mix design to achieve a balance between these properties. Single-factor experiments and an L_9_(3^3^) orthogonal array test were employed to evaluate the effects of target porosity (14–26%), water–cement ratio (0.26–0.34), sand rate (0–10%), and VMA dosage (0–0.02%). Additionally, Spearman rank correlation analysis and nonlinear regression fitting were utilized to develop quantitative relationships correlating the measured porosity to material performance. The results revealed that increasing target porosity enhances permeability but reduces compressive and splitting tensile strengths. The optimal water-to-cement ratio (w/c) was found to be 0.32, balancing both permeability and strength. An appropriate sand content of 6% improved mechanical properties, while a VMA dosage of 0.01% effectively enhanced bonding strength and workability. The orthogonal experiment identified the optimal mix ratio as a w/c ratio of 0.3, VMA dosage of 0.12%, target porosity of 14%, and sand content of 7%, achieving a compressive strength at 28-days of 43.5 MPa and a permeability coefficient of 2.57 mm·s^−1^. Empirical relationships for the permeability coefficient and mechanical properties as functions of the measured porosity were derived, demonstrating a positive exponential correlation between the measured porosity and the permeability coefficient, and a negative correlation with compressive and splitting tensile strengths. This research provides a systematic framework for designing high-performance pervious concrete with balanced permeability and mechanical properties, offering valuable insights for its development and application in green infrastructure projects.

## 1. Introduction

Pervious concrete, alternatively termed porous concrete, represents a specialized category of lightweight concrete designed with a substantial volume of interconnected voids that facilitate the efficient transmission of water and air [1,2]. This material is primarily composed of hydraulic cement as the binding agent, coarse aggregate providing the structural skeleton, and water, and often incorporates high-range water-reducing admixtures (HRWRAs) to enhance workability [3,4]. Distinguished from conventional impervious concrete by its minimal or negligible fine aggregate content, pervious concrete achieves a porous framework wherein coarse aggregates are coated with a thin layer of cement paste, creating an extensive network of permeable channels [5]. This distinctive architectural configuration confers exceptional permeability characteristics, rendering it particularly suitable for applications in sustainable drainage systems, permeable pavements, parking lots, and other urban surfaces where stormwater management, aquifer recharge, and mitigation of urban heat island effects are paramount [6,7,8].

The most critical characteristic defining pervious concrete is its designed porosity, which typically ranges from 15% to 30%, representing a compromise between hydraulic functionality and structural adequacy. This intentional void structure, however, establishes a well-recognized inverse relationship between permeability and mechanical strength, presenting a fundamental challenge in mixture proportioning and optimization [9,10]. The essential attributes, governing factors, and prevalent limitations associated with pervious concrete are systematically categorized in Table 1.

The mechanical performance of pervious concrete is predominantly governed by point-to-point contact mechanisms between aggregate particles [11]. The systematic exclusion of fine aggregates produces a structural matrix highly dependent on the integrity of the interfacial transition zone (ITZ) between the cement paste coating and coarse aggregates [12]. Consequently, the rheological behavior of the cementitious paste assumes critical importance in achieving microstructural homogeneity and overall composite stability. Suboptimal paste rheology manifests in two primary failure modes: excessive viscosity, resulting in inadequate workability, non-uniform coating, and compromised bonding; and insufficient viscosity, leading to particle segregation during placement, paste drainage, and stratified porosity distribution [13,14]. These phenomena underscore the necessity of precise regulation of mixture parameters, including w/c, superplasticizers, and viscosity-modifying agents (VMAs) [15,16,17].

The w/c ratio exerts profound influence on both fresh and hardened properties. Extremely low ratios (<0.26) may lead to incomplete hydration and deficient bonding strength, while excessively high ratios (>0.32) tend to improve workability at the expense of increased segregation susceptibility and reduced mechanical performance [18,19]. VMAs are frequently employed to enhance cohesive properties and mitigate segregation tendencies, thereby promoting uniform aggregate coating and improving resultant mechanical characteristics [20,21]. However, excessive dosage may impair workability and introduce undesirable air void systems, necessitating precise dosage optimization.

The incorporation of fine aggregates remains a subject of ongoing investigation. While conventional mixture designs exclude fines to preserve porosity, emerging research indicates that controlled incorporation can enhance mechanical properties without catastrophic permeability reduction [22,23]. Liu et al. [24] documented a 23.56% enhancement in compressive strength with 7% fine aggregate incorporation, accompanied by moderate pore structure modifications. Additional investigations have demonstrated improvements in abrasion resistance, sulfate resistance, and surface characteristics through partial fine aggregate inclusion [25,26,27]. Nevertheless, consensus confirms that permeability decreases proportionally with increasing fines content [28], emphasizing the necessity of balanced mixture design.

Although previous investigations have yielded valuable insights through single-factor experimental approaches, the complex synergies among multiple mixture parameters remain inadequately characterized. The coupled effects of target porosity, sand rate, w/c ratio, and admixture dosage lack comprehensive quantitative description. Furthermore, practical mixture design under multi-objective constraints—requiring simultaneous optimization of permeability and strength—demands enhanced understanding of factor interactions. Contemporary research has begun addressing these complexities through advanced statistical and experimental design methodologies. Representative studies have utilized factorial design and analysis of variance (ANOVA) techniques to identify significant parameters influencing pervious concrete performance [29,30,31]. Other investigators have employed correlation analyses to establish relationships between porosity, aggregate characteristics, and mechanical–hydraulic properties [32,33]. Despite these advances, a comprehensive methodology integrating both single-factor and multi-factor analyses to provide practical guidance for mixture proportioning remains underdeveloped.

To address these research deficiencies, this investigation systematically examines the individual and interactive effects of four pivotal mixture parameters—target porosity, sand rate, w/c ratio, and VMA dosage—on the compressive strength, splitting tensile strength, and permeability coefficient of pervious concrete. An integrated experimental strategy employing single-factor tests and L_9_(3^3^) orthogonal array testing was implemented. Range analysis and ANOVA facilitate assessment of parameter significance and interaction effects, while correlation analysis establishes quantitative relationships between porosity and engineering properties. It must be emphasized that, owing to the constrained dataset obtained in this study, the development of predictive models was intentionally avoided; instead, analytical emphasis was placed on interpreting factor effects and identifying robust correlations to inform practical mixture design. The findings of this research are expected to advance fundamental understanding of parameter interactions in pervious concrete, thereby supporting the development of optimized mixture designs for enhanced performance in sustainable infrastructure applications.

## 2. Materials and Methods

### 2.1. Raw Materials

#### 2.1.1. Cement

This experiment utilized P·II 52.5R grade Portland cement produced by the Conch Cement Plant in Ma’anshan, Anhui Province, China. Its specific surface area was 338.70 m^2^/g, fineness (residue on 0.08 mm sieve) was 0.32%, initial setting time was 180 min, and final setting time was 230 min.

#### 2.1.2. Coarse Aggregate

The coarse aggregate used in this study was basalt crushed stone with a particle size of 5–10 mm. Its fundamental physical properties, tested according to GB/T14685-2011, “Pebble and crushed stone for construction,” were as follows: apparent density of 2941 kg/m^3^, bulk density of 1656 kg/m^3^, and porosity of 43.69%.

#### 2.1.3. Fine Aggregate

To achieve the synergistic optimization goal of “strength–permeability,” river sand subjected to secondary washing and sieving was selected to minimize the adverse effects of harmful impurities on ITZ bonding. According to GB/T 14684-2022, “Sand for construction,” the fineness modulus of the river sand was 2.7, classifying it as medium sand in Zone II.

#### 2.1.4. Viscosity-Modifying Admixture (VMA)

VMA1-type viscosity modifier (white powder, drying loss 2.3%, pH ≈ 7.0, molecular weight Mw ≈ 2.0 × 10^6^ g/mol) produced by Nanjing Pingda Engineering Co., Ltd. (Nanjing, China). was selected as the main experimental variable. Its carboxymethyl ether groups exhibit moderate chelation with free Ca^2+^, delaying early heat of hydration and reducing internal stress associated with the temperature peak while maintaining fluidity.

#### 2.1.5. Water-Reducing Agent

The polycarboxylate-based superplasticizer (JC-H) used in this study was self-developed by the research group. It exhibits high water-reducing and slump-retaining capabilities, with a solid content of 40%. Its performance complies with the GB8076-2008 “Concrete admixtures” standard.

### 2.2. Mix Proportion Design

#### 2.2.1. Single-Factor Mix Proportion Design

Single-factor experiments were conducted to systematically investigate the effects of four key mix parameters—target porosity, water–cement (w/c) ratio, sand rate, and viscosity-modifying admixture (VMA) dosage—on the permeability and mechanical properties of pervious concrete. For all single-factor groups, specimens were prepared volumetrically and tested for the permeability coefficient, actual porosity, compressive strengths at 7-days and 28-days, and splitting tensile strength at 28-days. The detailed mix proportions for all single-factor groups are summarized in Table 2.

Permeability is a crucial indicator for pervious concrete. While meeting strength requirements, the concrete must also achieve a certain permeability coefficient. During mix design, permeability cannot be directly reflected, whereas porosity directly reflects the structure of pervious concrete and significantly impacts its performance. Furthermore, the design and selection of target porosity directly affect the actual internal pore structure. Target porosities of 14%, 17%, 20%, 23%, and 26% were designed. The detailed calculation process of the target porosity and the correlation analysis between the target porosity and the measured porosity are shown in Appendix A.

The water–cement (w/c) ratio significantly influences the permeability of pervious concrete. W/c ratios that are too high or too low affect paste fluidity and the distribution of internal pores, thereby altering macroscopic performance. Five w/c ratios (0.26, 0.28, 0.30, 0.32, 0.34) were designed for specimens with a target porosity of 20%.

Incorporating an appropriate amount of fine aggregate can improve pervious concrete performance. However, adding fine aggregate fills voids between aggregates, inevitably affecting permeability. Sand replacement ratios of 0%, 2%, 4%, 6%, 8%, and 10% were used for specimens with a target porosity of 20%.

Viscosity-modifying agent (VMA) was incorporated into pervious concrete (target porosity 20%) at dosages of 0, 0.005%, 0.01%, 0.015%, and 0.02%. VMA is a water-soluble polymer additive that delays cement hardening and setting rates and critically influences the thickening and water-retention properties of the concrete paste.

#### 2.2.2. Orthogonal Test

To prepare high-strength pervious concrete, based on the results of the single-factor experiments, an orthogonal test design (L_9_(3^3^)) was employed, selecting target porosity, sand rate, and VMA dosage as the three factors. The w/c ratio was fixed at 0.3, and the JC-H superplasticizer dosage at 0.12%. Sand rate levels were 4%, 6%, and 8%; VMA dosage levels were 0.005%, 0.01%, and 0.015%; target porosity levels were 14%, 20%, and 26%. Specimens were prepared volumetrically. The factor level table is shown in Table 3, and the orthogonal test layout L_9_(3^3^) is shown in Table 4.

### 2.3. Sample Preparation and Curing

In this experiment, a mixing method involving coating the aggregates with cement paste was employed. This approach ensures uniform distribution of the mixture and complete coverage of aggregates by the cement paste, significantly enhancing inter-aggregate bonding and consequently improving the physical strength of the pervious concrete. First, 50% of the mixing water was added to the aggregates and mixed for 30 s. After the aggregate surfaces became moist, cement and other cementitious materials were added and mixed for another 60 s. Once the cementitious materials were uniformly mixed with the aggregates, the remaining 50% of water and the superplasticizer were gradually added. Mixing continued for 60 s before discharging the mixture.

To prevent excessive vibration from causing cement paste sedimentation and blocking pores, thereby affecting permeability, a combination of manual rodding and machine vibration was used for compaction. Fresh concrete was placed into the molds in three layers, with each layer compacted. The molds were then placed on a vibrating table and vibrated twice: the first vibration lasted 10 s, and the second lasted 5 s. Finally, the top surface was leveled. After molding, specimens were covered with plastic film. Demolding was performed after 24 h, and the specimens were subsequently placed in a standard curing room for further curing and testing.

### 2.4. Characterization of Performance

#### 2.4.1. Compressive Strength Test

Standard cubic specimens of size 100 mm × 100 mm × 100 mm were used. Compressive strength testing was conducted according to “Standard for test method of mechanical properties on ordinary concrete” (GB/T 50081-2016) using a YE-1000 hydraulic pressure testing machine. The loading rate was controlled between 1000–3000 N/s. The compressive strength (*f*) was calculated using Equation (1):

(1) $$f=\frac{F}{A}$$

where *F* is the maximum load at failure (N), and *A* is the cross-sectional area of the specimen (mm^2^).

#### 2.4.2. Splitting Tensile Strength Test

Splitting tensile strength was tested using the YE-1000 hydraulic pressure testing machine according to “Standard for test methods of concrete physical and mechanical properties” (GB/T 50081-2019). Cubic specimens (100 mm × 100 mm × 100 mm) were used. Three specimens were tested per group, and the average value was taken.

#### 2.4.3. Permeability Testing

**Figure 1:**
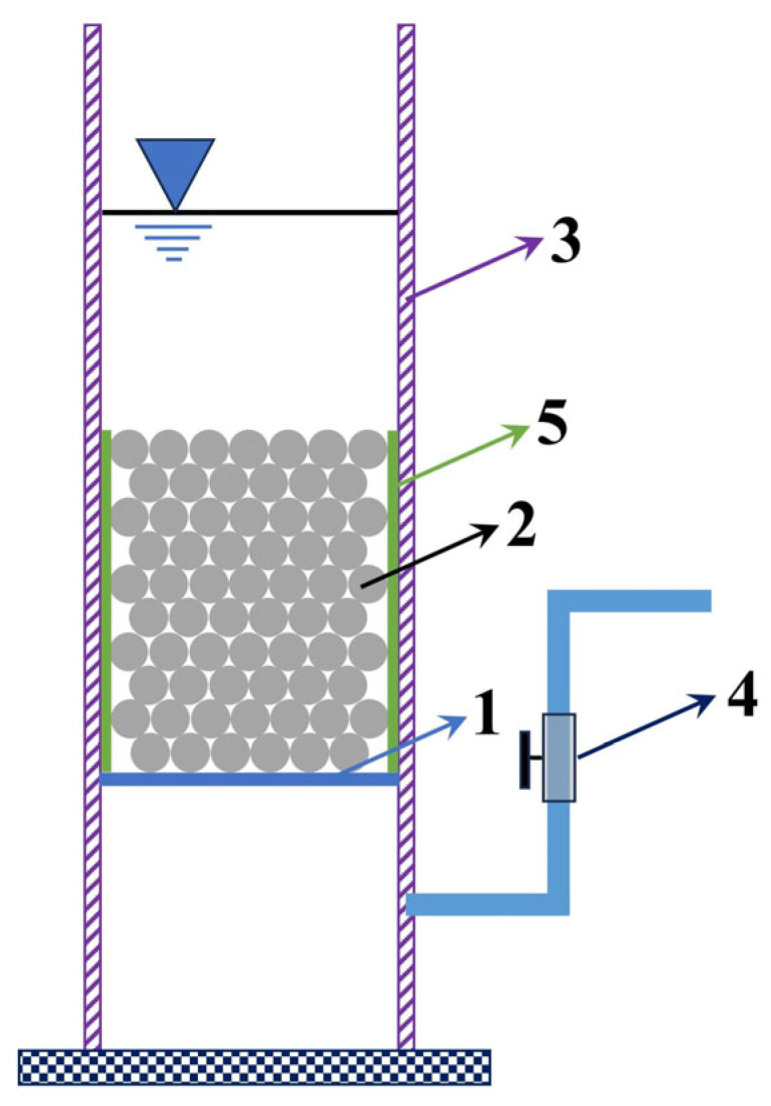
Schematic diagram of the falling head permeability test apparatus. 1: Filter mesh (also supporting the specimen), 2: Specimen, 3: Cylindrical wall (plexiglass), 4: Water stop valve, 5: Plastic wrap layer (ensuring tight contact between specimen and wall to prevent side leakage).

Cylindrical specimens (D = 70 mm, H = 150 mm) cast in PVC pipes (Figure 1) were used for permeability tests. A “falling head” method was employed to determine the permeability coefficient using a custom-built permeameter. The permeability coefficient (*k*) was calculated using Equation (2):

(2) $$k=\frac{aL}{At}\ln\left(\frac{h_{1}}{h_{2}}\right)$$

where *L* is the specimen height (mm), *A* is the specimen cross-sectional area (mm^2^), *a* is the cross-sectional area of the standpipe (mm^2^), *h*_1_ and *h*_2_ are the initial and final water head levels (mm), respectively, and *t* is the time taken for the head to fall from *h*_1_ to *h*_2_ (s).

## 3. Results and Discussion

### 3.1. Single-Factor Analysis

#### 3.1.1. Effect of Target Porosity

**Figure 2:**
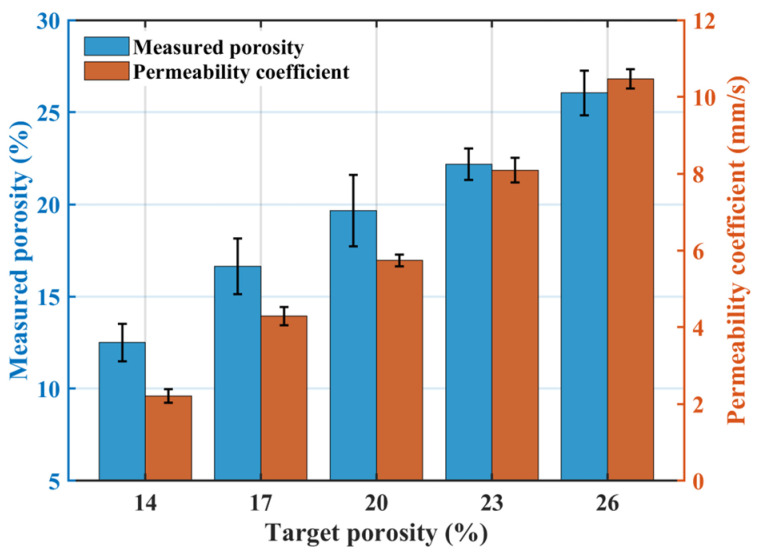
Effect of target porosity on the permeability coefficient.

Figure 2 illustrates the effect of target porosity on permeability: both the permeability coefficient and the measured porosity of pervious concrete increase with increasing target porosity. The measured porosities for groups TP1–TP5 were 12.50%, 16.63%, 19.66%, 22.18%, and 26.05%, respectively, closely matching the designed target porosities. This confirms the applicability of the volumetric method for pervious concrete mix design. However, target porosity values were consistently higher than the measured porosity. This discrepancy arises because the measured porosity excludes closed and semi-connected pores present within the concrete [34]. Additionally, achieving absolute compaction between cement paste and aggregates during preparation is difficult, resulting in the measured porosity being lower than the target value.

**Figure 3:**
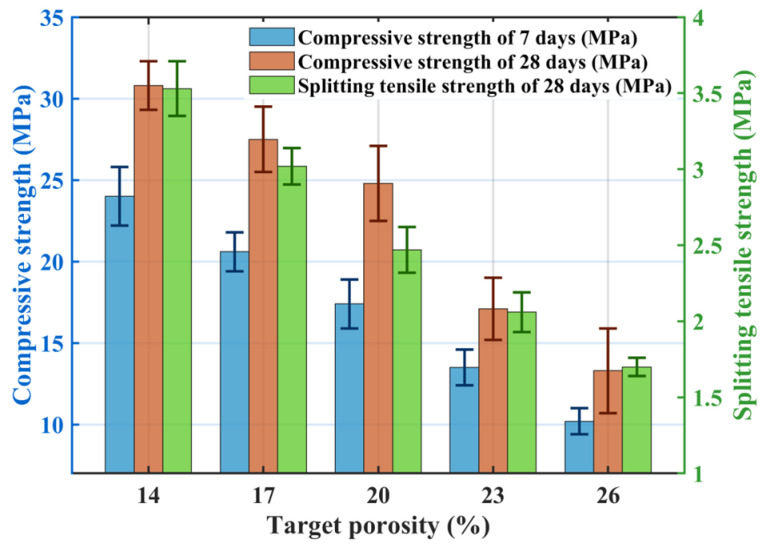
Effect of target porosity on mechanical properties (7 d CS, 28 d CS, STS).

Figure 3 shows the effect of target porosity on mechanical properties: both compressive strength and splitting tensile strength decrease as target porosity increases. The TP1 group achieved compressive strengths at 7-days and 28-days of 24.1 MPa and 30.8 MPa, respectively, with a splitting tensile strength of 3.53 MPa. Compared to TP1, the TP5 group exhibited reductions of 57.2% and 56.5% in compressive strength at 7-days and 28-days, respectively, and a 51.8% reduction in splitting tensile strength. Pervious concrete is an integrated structure composed of pores and cement paste-coated coarse aggregates. Its compactness is a key factor influencing mechanical properties. As target porosity increases, internal pores increase, reducing overall compactness. Increased porosity enlarges the distance between aggregates, decreases their contact area, and weakens bonding [35]. The weakest points in pervious concrete are typically the interfacial layers between cement paste and coarse aggregates at pore locations, which are more prone to cracking under stress [36]. As internal pores increase, more weak points exist, making stress concentration more likely to initiate cracks, leading to reduced mechanical performance.

#### 3.1.2. Effect of Water–Cement Ratio

**Figure 4:**
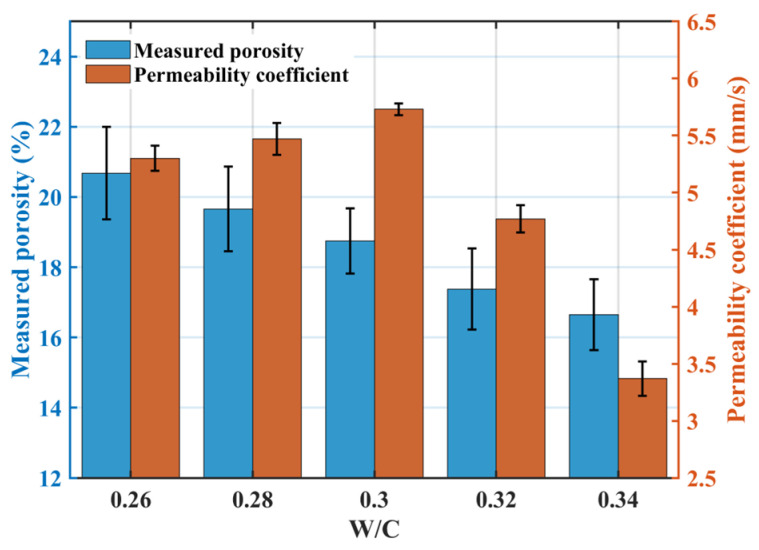
Effect of the w/c ratio on the permeability coefficient.

Figure 4 illustrates the effect of the w/c ratio on permeability: the measured porosity decreased with increasing w/c ratio, while the permeability coefficient initially increased and then decreased, peaking at a w/c ratio of 0.30 (5.7 mm·s^−1^). When the w/c ratio exceeded 0.30, increased paste fluidity led to downward sedimentation during mixing, causing a rapid decline in the permeability coefficient.

**Figure 5:**
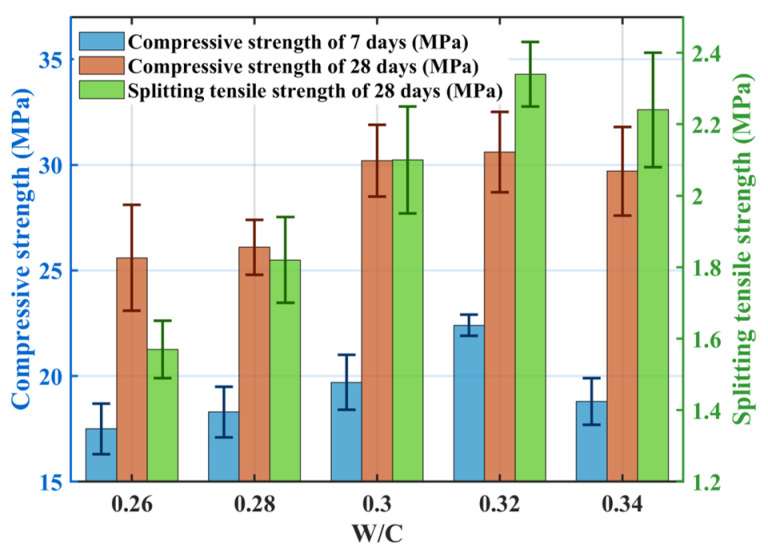
Effect of the w/c ratio on the mechanical properties (7 d CS, 28 d CS, STS).

Figure 5 shows the effect of the w/c ratio on mechanical properties: both compressive and splitting tensile strengths initially increased within the w/c range of 0.26–0.32, reaching maximum values at a w/c ratio of 0.32. At this ratio, the compressive strengths at 7-days and 28-days were 22.4 MPa and 30.6 MPa, respectively, and the splitting tensile strength was 2.34 MPa. When the w/c ratio was too low (<0.32), insufficient water hindered complete cement hydration, limiting C-S-H gel formation [37]; unhydrated cement particles contributed little strength. Furthermore, low paste fluidity and high static yield stress hindered mixing, resulting in uneven aggregate coating and poor bonding [13]. At the optimal w/c ratio of 0.32, static yield stress decreased, paste fluidity increased, and mixture workability improved, facilitating uniform paste coating on aggregates and strong bonding. Concurrently, internal void distribution became more uniform, strengthening weaker zones. When the w/c ratio exceeded 0.32, although cement particles might hydrate fully, increased paste fluidity drastically reduced plastic viscosity and static yield stress. This caused paste sedimentation in the upper part of the specimens, uneven internal paste distribution, insufficient paste coating on aggregates, reduced aggregate connectivity, and the formation of large voids and microcracks in the upper sections, ultimately reducing strength.

#### 3.1.3. Effect of Sand Rate

**Figure 6:**
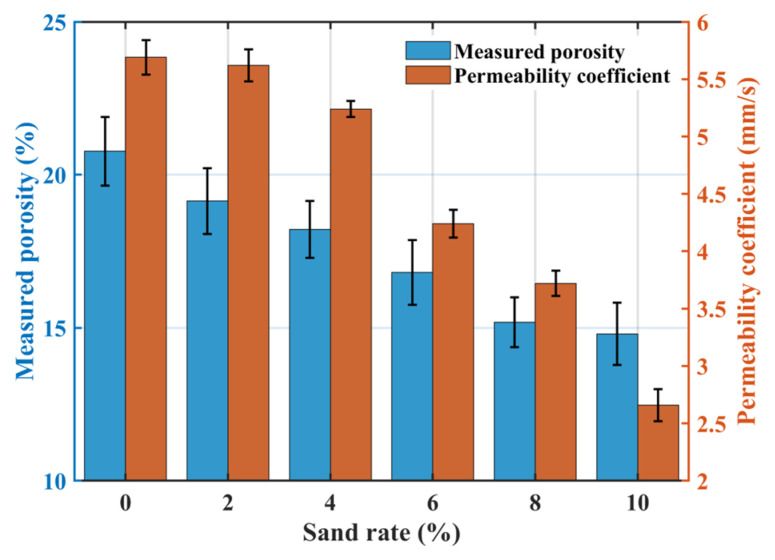
Effect of sand rate on the permeability coefficient.

Figure 6 illustrates the effect of sand rate on permeability: both the measured porosity and the permeability coefficient decrease with increasing sand rate. At a sand rate of 10%, the measured porosity was only 14.77%, and the permeability coefficient dropped to 1.85 mm·s^−1^. This occurs because sand replaces part of the coarse aggregate volume, and its incorporation increases the paste volume, refines internal voids, and improves the density of the interfacial transition zone, thereby reducing permeability.

**Figure 7:**
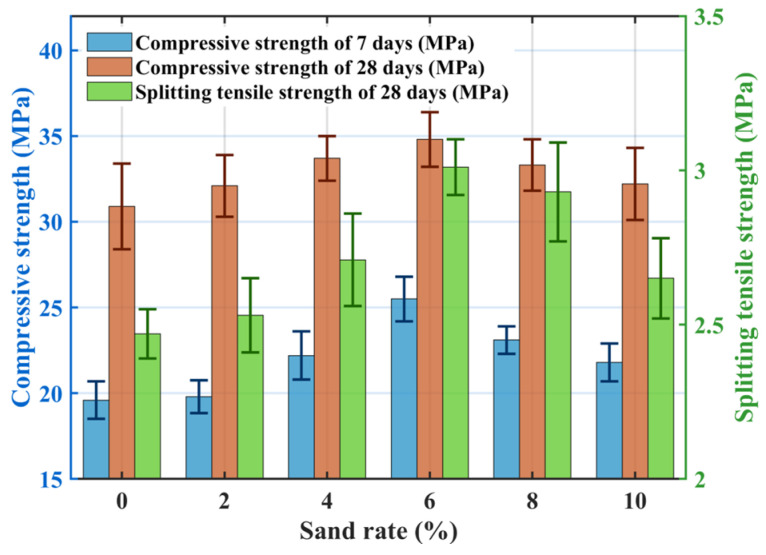
Effect of sand rate on the mechanical properties.

Figure 7 shows the effect of sand rate on mechanical properties: compressive and splitting tensile strengths initially increase and then decrease with increasing sand rate. At a sand rate of 6%, the compressive strengths at 7-days and 28-days increased by 30.7% and 12.6%, respectively, compared to the no-sand mix (SR0), and splitting tensile strength increased by 21.9%. Incorporating a small amount of fine sand to replace part of the coarse aggregate fills voids within the skeletal pore structure of pervious concrete, enhancing the density of the interfacial transition zone and improving overall packing. Simultaneously, a mixed layer of fine sand and cement forms on the coarse aggregate surface, creating a bonding layer between particles that increases concrete stiffness. During mechanical testing, cracks propagating from the surface inward are impeded by the presence of fine sand, requiring greater energy to destroy or bypass these positions, thus improving mechanical properties. However, excessive sand rate (>6%) leads to decreased mechanical performance. Wetting the surface of fine aggregates consumes mixing water. High sand rates increase water demand for wetting, reducing water available for cement hydration. Moreover, fine sand particles, being larger than cement particles, hinder particle movement, reducing the fluidity of the fresh mixture and increasing mixing difficulty [38]. Additionally, high sand rates can lead to insufficient cement paste to fully coat the fine sand surfaces, resulting in a looser mixture with fewer contact points between aggregates, thereby reducing strength.

#### 3.1.4. Effect of VMA Dosage

**Figure 8:**
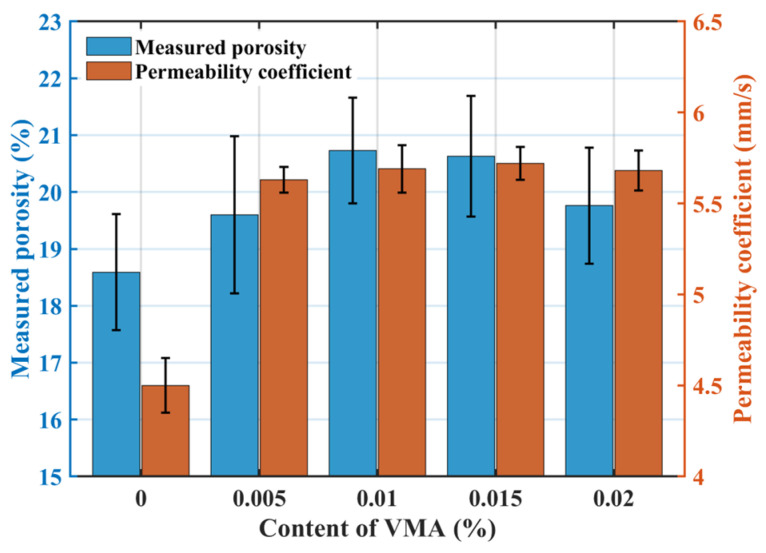
Effect of VMA dosage on the permeability coefficient.

Figure 8 illustrates the effect of VMA dosage on permeability: the measured porosity initially increased slightly and then decreased with increasing VMA dosage, while the permeability coefficient increased initially and then stabilized. The target porosity was 20%, and the measured porosity for the VMA groups fluctuated between 18.64% and 22.8%, consistent with the design. VMA incorporation does not significantly alter the skeletal structure. VMA improves paste workability, preventing sedimentation and blockage, thus increasing permeability at dosages of 0.005% and 0.01%. Further increases in VMA dosage had negligible impact on permeability.

**Figure 9:**
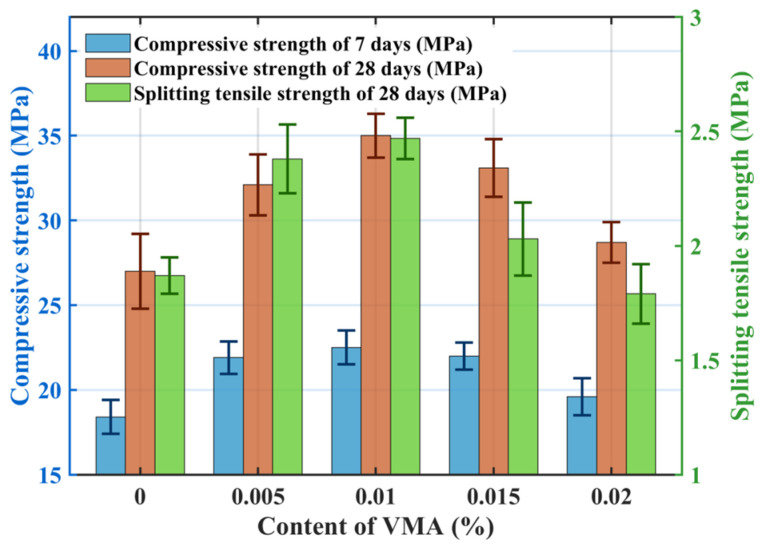
Effect of VMA on the mechanical properties.

Figure 9 shows the effect of VMA dosage on mechanical properties: both compressive and splitting tensile strengths initially increase and then decrease with increasing VMA dosage. Optimal mechanical performance was achieved at a VMA dosage of 0.01% (VMA3), with compressive strengths at 7-days and 28-days reaching 22.5 MPa and 34.9 MPa, respectively, and splitting tensile strength reaching 2.47 MPa. Strength decreased when VMA dosage exceeded 0.01%, although it remained higher than that of the control group (VMA1). Groups VMA4 and VMA5 exhibited compressive strength at 28-days reductions of 6.1% and 22.0%, respectively, compared to VMA3. Incorporating VMA increases solution viscosity. As dosage increases, the intertwining of VMA and cement paste during mixing alters hydration, increasing paste viscosity and yield stress. This hinders aggregate movement, promoting tight paste coating and bonding after setting. Appropriate VMA optimizes internal pore structure, forming a network bonded to hydration products, densifying the internal structure. Additionally, increased VMA improves mixture workability, enhancing mechanical properties. However, excessive VMA dosage leads to very high paste viscosity, increasing mixing difficulty and causing uneven paste coating on coarse aggregates. Furthermore, the high molecular weight and air-entraining/thickening effects of VMA introduce stable air bubbles that are difficult to expel due to increased viscosity. These bubbles create internal defects within the paste, progressively reducing concrete strength.

### 3.2. Orthogonal Test Results Analysis and Mix Optimization

#### 3.2.1. Permeability Coefficient: Range Analysis and Contour Mapping

The permeability coefficient (*K*, mm/s) is a critical indicator of the water transport capacity of pervious concrete, influenced by the interactive effects of sand rate, viscosity-modifying admixture (VMA) dosage, and target porosity. The L_9_(3^3^) orthogonal test, range analysis, analysis of variance (ANOVA), and contour mapping were employed to evaluate the significance and coupling effects of these factors, providing a theoretical foundation for designing highly permeable pervious concrete.

The orthogonal test results (Table 5) revealed a wide variation in permeability coefficients, ranging from 2.4 to 9.1 mm·s^−1^ (OED9 to OED3), reflecting a high sensitivity to mix parameters. A strong positive correlation was observed between porosity and permeability: specimens with 26% porosity (high level, C3) consistently exhibited coefficients exceeding 8.5 mm·s^−1^, with OED3 (4% sand rate) achieving the maximum value of 9.1 mm·s^−1^. Conversely, specimens with 14% porosity (low level, C1) all had coefficients below 4.6 mm·s^−1^, with OED9 recording the minimum value of 2.4 mm·s^−1^.

Range analysis (Table 6) quantified the influence of each factor: the range for target porosity (R_C_ = 5.07 mm·s^−1^) substantially exceeded those for sand rate (R_A_ = 1.13 mm·s^−1^) and VMA dosage (R_B_ = 0.57 mm·s^−1^). As porosity increased from 14% to 26%, the average permeability coefficient rose from 3.70 to 8.7 mm·s^−1^—a 137% increase. This increase, though significant, was less than the theoretical prediction based on the Hagen–Poiseuille law (which suggests a 245% increase for an 86% rise in porosity), likely due to the non-ideal connectivity and tortuosity of actual pores.

The influence of sand rate manifested as a gradual attenuation of permeability. For instance, at 26% porosity, increasing the sand rate from 4% (OED3) to 8% (OED8) reduced permeability from 9.1 to 8.5 mm·s^−1^. However, at low porosity (14%), the same increase in sand rate led to a sharp decline in permeability (e.g., from 4.6 to 2.4 mm·s^−1^ for OED1 to OED9). Fine sand particles do not directly clog the pore channels but primarily occupy the void spaces within the aggregate skeleton, thereby reducing the effective porosity and lowering permeability. This mechanism explains why even small additions of fine sand can markedly diminish hydraulic conductivity, particularly under low target porosity conditions. From a fluid mechanics perspective, the Hagen–Poiseuille law describes flow through idealized capillary tubes, predicting that the permeability coefficient is highly sensitive to pore diameter (proportional to the fourth power). When fine sand reduces the effective pore size and continuity, the actual increase in flow resistance is much more pronounced than the reduction in porosity alone would suggest [39]. Therefore, the experimental results, showing a steep decline in permeability with higher sand contents, are consistent with theoretical expectations from pore-scale flow laws. VMA dosage exhibited the weakest influence, with permeability varying by less than 10% across its levels.

ANOVA results (Table 7) further confirmed the dominance of target porosity, which yielded a highly significant F-value of 86.66. In contrast, sand rate (F = 4.23) and VMA (F = 1.23) were not statistically significant. The optimal combination for maximizing permeability was identified as A1B1C3 (sand rate 4%, VMA 0.015%, porosity 26%).

**Figure 10:**
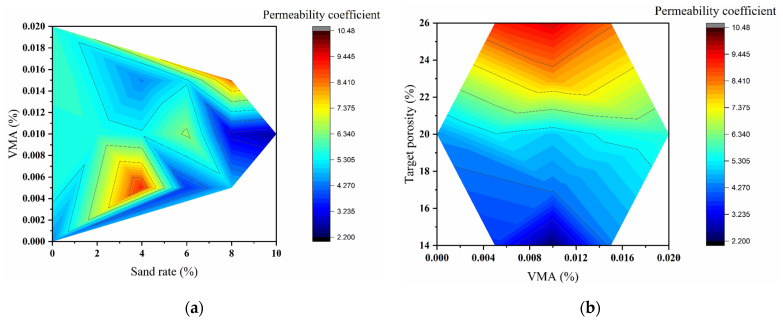
Contour maps of the permeability coefficient: (**a**) Sand rate vs. VMA dosage; (**b**) VMA dosage vs. target porosity.

Contour maps (Figure 10) visualized the coupling effects between parameters. Figure 10a shows that high permeability (>8.0 mm·s^−1^) was achieved at low sand rates (3–5%) and moderate VMA dosages (0.04–0.06%). Beyond 5% sand rate, permeability decreased gradiently due to pore blockage. Figure 10b illustrates that permeability increased markedly once porosity exceeded 22%, while varying VMA dosage had negligible impact—consistent with ANOVA results.

In summary, target porosity is the predominant factor controlling permeability, followed by sand rate, while VMA plays a minor role. These findings provide clear guidance for designing pervious concrete with tailored hydraulic performance.

#### 3.2.2. Mechanical Properties: Range Analysis and Contour Mapping

The mechanical properties of pervious concrete, including compressive strength at 7-days (7 d CS), compressive strength at 28-days (28 d CS), and 28-day splitting tensile strength (STS), were evaluated using the L_9_(3^3^) orthogonal array. Range analysis and analysis of variance (ANOVA) was employed to assess the influence and significance of three key factors—target porosity, sand rate, and VMA dosage. Contour maps were further utilized to visualize the coupling effects between these parameters.

The range analysis results are presented in Table 8. For 7 d CS, the range values (R) were as follows: Target Porosity = 12.91 MPa, Sand Rate = 8.20 MPa, VMA = 4.09 MPa. This indicates that target porosity had the greatest influence on early strength, followed by sand rate and VMA dosage. For 28 d CS, the range values were Target Porosity = 12.93 MPa, Sand Rate = 11.17 MPa, VMA = 3.03 MPa. Although target porosity remained the most influential factor, the effect of sand rate increased compared to the 7-day results, suggesting a more pronounced role of the aggregate skeleton in long-term strength development. In the case of STS, the range values were Target Porosity = 0.42 MPa, Sand Rate = 0.36 MPa, VMA = 0.28 MPa. Despite smaller numerical values, the relative influence hierarchy remained consistent, with porosity being the most significant factor. This underscores the particular sensitivity of tensile properties to internal pore structure and interfacial bonding quality. Across all mechanical properties, target porosity consistently emerged as the dominant factor. The optimal parameter combinations frequently included low target porosity (14%), often in combination with higher sand rate (8%), indicating a synergistic effect between these two factors.

ANOVA was performed to evaluate the statistical significance of each factor (Table 9, Table 10 and Table 11). For 7 d CS, target porosity showed high significance (F = 17.93), while sand rate (F = 7.43) and VMA (F = 1.74) were not statistically significant at α = 0.05. For 28 d CS, both target porosity (F = 17.91) and sand rate (F = 13.42) were statistically significant, while VMA remained non-significant (F = 1.00). This confirms the increasing importance of aggregate packing characteristics for later-age compressive strength. For STS, all three factors showed high significance: Target Porosity (F = 303.25), Sand Rate (F = 89.75), and VMA (F = 28.50). The exceptional significance values highlight the complex relationship between pore structure, aggregate gradation, and paste properties in determining tensile performance.

**Figure 11:**
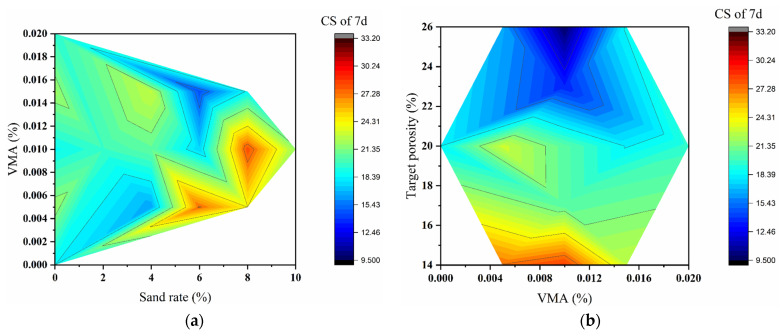

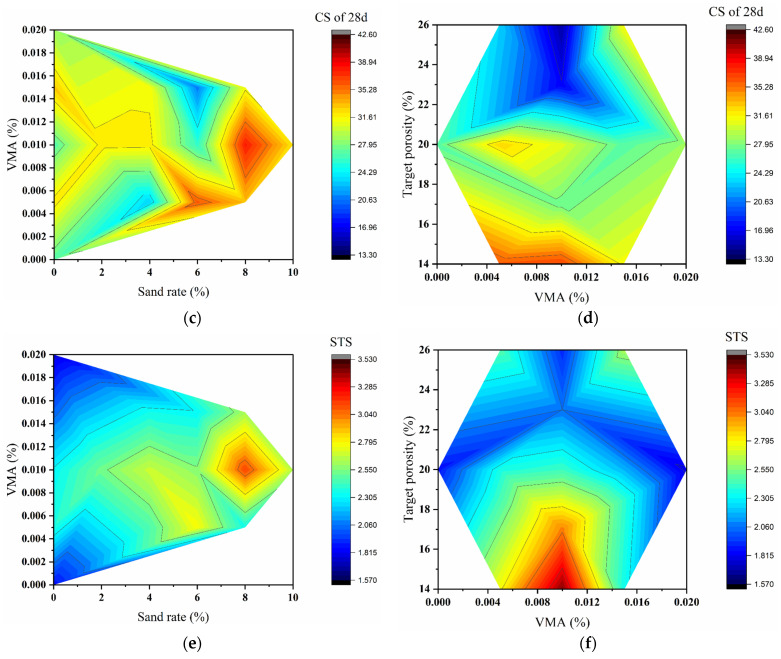
Contour maps of mechanical properties vs. sand rate and target porosity (**a**–**c**) and 3D response surfaces vs. sand rate and VMA dosage (**d**–**f**). (**a**,**d**) 7 d CS; (**b**,**e**) 28 d CS; (**c**,**f**) STS. (VMA dosage fixed at a specific level for contour maps; target porosity fixed at a specific level for response surfaces.)

Contour maps (Figure 11a–c) illustrate the combined effects of target porosity and sand rate on mechanical properties. Figure 11a (7 d CS) shows that the highest strength values occur in regions of low-to-medium porosity (14–20%) and moderate sand rate (4–6%). Strength decreases significantly when porosity exceeds 20% or sand rate reaches 8%, creating apparent “low-strength valleys.” Figure 11b (28 d CS) reveals smoother contour lines, indicating reduced sensitivity to local mix variations with ongoing hydration. The optimal strength region remains in the low-to-medium porosity range (14–20%), with distinct high-strength zones appearing at specific sand rate values. Figure 11c (STS) demonstrates a more complex response pattern. The highest tensile strength is achieved at low porosity (14–20%) and low-to-medium sand rate (4–6%). A sharp decline occurs when porosity reaches 26%, emphasizing the critical importance of structural integrity for tensile performance. The contour analysis consistently demonstrates that target porosity is the primary factor governing mechanical properties, while the sand rate shows increasing influence with concrete age. The interactive effects between these factors create distinct regions of optimal performance, particularly in the range of 14–20% porosity and 4–8% sand rate.

In summary, contour maps effectively revealed the decisive influence of target porosity across all mechanical properties and the relative importance and synergy of sand rate and VMA for different performance indicators. For STS, the complex non-linear coupling among the three factors was visualized. Identifying optimal mix regions, especially the “performance plateaus” and “sensitive transition zones,” provides a visual risk assessment framework for mix design. The optimal combination A3B1C1 (Sand rate 8%, VMA 0.015%, Porosity 14%), located at the convergence of favorable performance zones for all three indicators, demonstrates good practical value.

#### 3.2.3. Mix Optimization and Verification

Based on the orthogonal test benchmark group A3B1C1 (Sand rate 8%, VMA 0.015%, Porosity 14%), and considering the sand rate as a significant influencing factor identified earlier, further optimization was conducted by selecting sand rates of 7%, 8%, and 9% to prepare high-strength pervious concrete. The optimization test series is denoted as YZ; mix proportions are shown in Table 12.

The experimental results are shown in Table 13: the mechanical properties of all YZ groups exceeded the highest values from the orthogonal test group. Thus, the A3B1C1 benchmark group (OED9 equivalent) was confirmed as the orthogonal test’s optimal group for mechanical performance. In the significance optimization test, at a sand rate of 7% (YZ1), 7-day and 28-day strengths were 5.1% and 8.1% higher than those of the orthogonal verification group (YZ0/OED9 equivalent), respectively, and splitting tensile strength was 0.6% higher. At a sand rate of 9% (YZ2), all mechanical properties were lower than those of the orthogonal verification group. The permeability coefficient of YZ1 reached 2.57 mm·s^−1^, satisfying pervious concrete requirements. Therefore, the YZ1 mix (Target Porosity 15%, w/c ratio 0.3, PCE 0.12%, VMA 0.01%, Sand Rate 7%) was identified as the optimal proportion for high-strength pervious concrete.

### 3.3. Correlation Analysis of Pervious Concrete

#### 3.3.1. Spearman Correlation Coefficient Analysis

Revealing the correlations between design parameters and material performance is fundamental for mix optimization and understanding performance trends in multi-performance regulation of pervious concrete. This study employed Spearman’s rank correlation coefficient (SCC) to systematically quantify the correlations between key design factors—porosity (design P_T_ and measured P_M_), water–cement (w/c) ratio, sand rate (SR), and VMA dosage—and major performance indicators: permeability coefficient (PC), compressive strength at 7-days (CS_7d_), compressive strength at 28-days (CS_28d_), and splitting tensile strength (STS). The analysis aimed to preliminarily identify factors with significant positive or negative regulatory effects on target performance, providing a basis for variable selection in predictive modeling.

The Spearman correlation coefficient was calculated using the following standard formula:

(3) $$\rho =1-\frac{6\sum d_{i}^{2}}{n\left(n^{2}-1\right)}$$

where *d_i_* is the difference between the ranks of each pair of corresponding values for the two variables. *n* is the number of paired observations (data points). The summation (∑) is taken over all *n* pairs.

**Figure 12:**
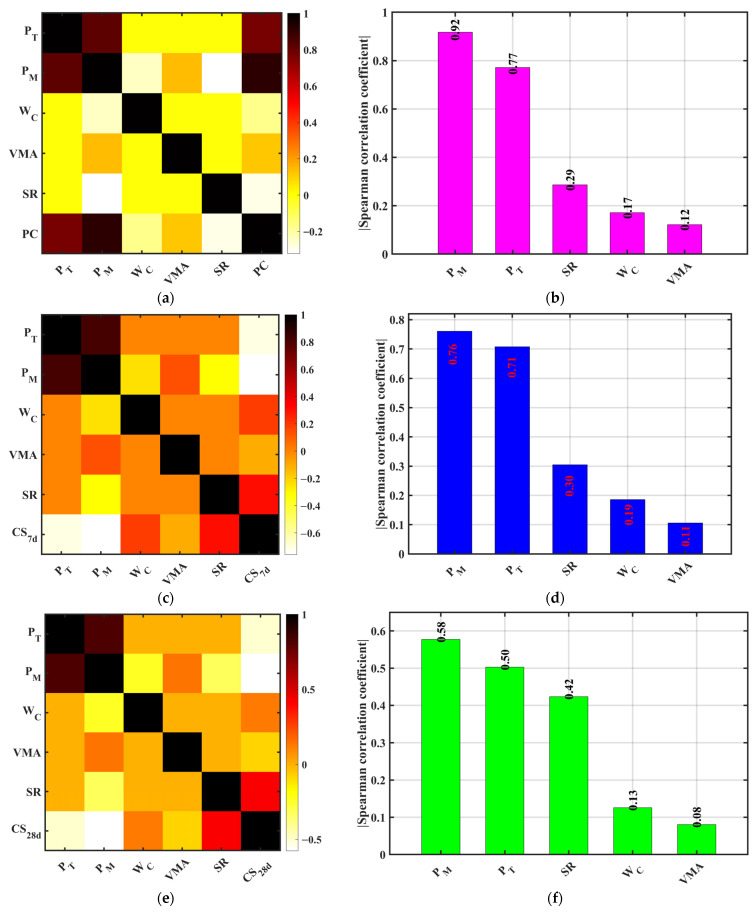

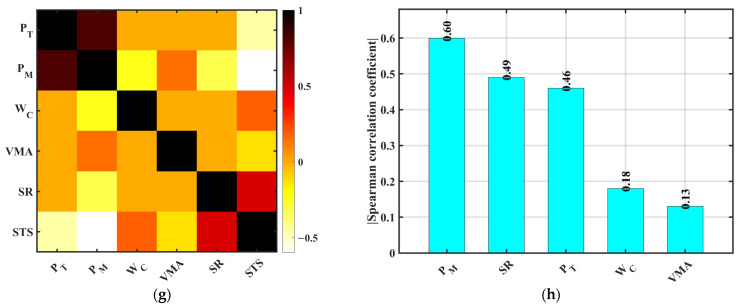
Spearman rank correlation coefficient matrices. (**a**,**c**,**e**,**g**) Correlation with design porosity (P_T_); (**b**,**d**,**f**,**h**) Correlation with measured porosity (P_M_). (**a**,**b**) Permeability coefficient (P_C_); (**c**,**d**) 7 d Compressive strength (CS_7d_); (**e**,**f**) 28 d Compressive strength (CS_28d_); (**g**,**h**) Splitting tensile strength (STS).

As shown in Figure 12, both P_M_ and P_T_ exhibit highly significant positive correlations with PC, with Spearman coefficient magnitudes of 0.92 and 0.77, respectively. This indicates that porosity (whether designed or measured) has the most direct and strongest influence on permeability. This aligns with previous theoretical and experimental studies demonstrating that porosity is the dominant parameter controlling permeability in pervious concrete [9,40]. Target porosity dictates void configuration during design, while the measured porosity reflects the actual void fraction achieved, both strongly coupled with PC. The measured porosity shows a higher correlation, suggesting that final performance depends more on construction quality than design specifications, highlighting the importance of process control and quality testing. In contrast, the w/c ratio and SR show weak negative correlations with PC (Spearman coefficients ≈ −0.17 and −0.29), consistent with material principles: a higher w/c ratio increases paste fluidity but may dilute hydration products and create discontinuous flow paths; higher SR tends to densify the skeleton, reducing porosity and limiting permeability. VMA shows the weakest correlation with PC (≈0.12), indicating its minor contribution to permeability, likely more related to mixture cohesiveness and segregation resistance than pore connectivity.

For compressive performance, P_M_ and P_T_ show significant negative correlations with CS_7d_ (Spearman coefficients ≈ −0.76 and −0.71), confirming that increased porosity significantly reduces early strength. Higher porosity means larger gaps between aggregates, reduced paste filling capacity, limited hydration product formation, poorer structural compactness, and lower strength. The w/c ratio and SR show positive correlations with CS_7d_ (≈0.19 and 0.30), with SR having a more pronounced effect, indicating appropriate aggregate proportions aid early strength development. VMA shows no significant correlation with CS_7d_. For CS_28d_, P_M_ and P_T_ maintain negative correlations (≈−0.58 and −0.50), slightly lower than at 7d but still significant. This may relate to partial pore filling by hydration products over time. The w/c ratio and SR remain positively correlated (≈0.13 and 0.42), with SR’s influence strengthening at 28 days, reflecting the increased role of the aggregate skeleton. VMA’s influence remains negligible. For STS, correlations align with compressive trends: P_M_ and P_T_ negatively correlated (≈−0.60 and −0.49), the w/c ratio and SR positively correlated (≈0.18 and 0.46). This fits the failure mechanism in which stress concentration and crack propagation relate to pore structure: higher porosity creates more microcrack paths, causing premature failure.

In summary, Spearman correlation analysis clearly reveals that the measured and design porosity are key factors influencing the permeability and mechanical properties of pervious concrete, with paramount significance for permeability control. The sand rate plays an important role in strength development, particularly at later ages; the w/c ratio contributes moderately; VMA shows weak influence on strength and permeability.

#### 3.3.2. Correlation Relationships

**Figure 13:**
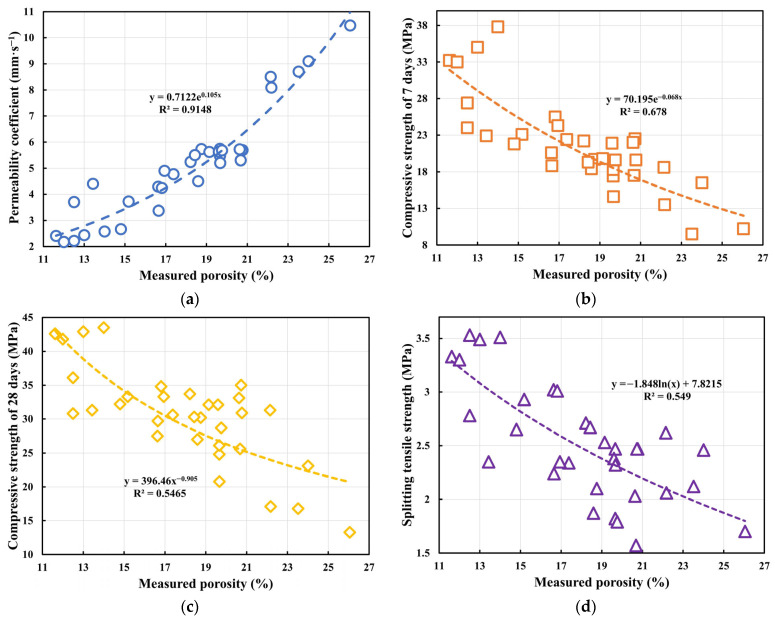
Fitted correlation relationships between the measured porosity and performance: (**a**) Permeability coefficient (PC); (**b**) 7-day compressive strength (CS_7d_); (**c**) 28-day compressive strength (CS_28d_); (**d**) Splitting tensile strength (STS).

Building on the identified parameter–performance correlations, regression fitting was used to explore the quantitative relationship between the measured porosity (*P_M_*) and key pervious concrete properties. Given the often non-linear (e.g., exponential, power, logarithmic) response of concrete properties to porosity changes, typical non-linear functions were fitted. Combined with the fitting results in Figure 13, the correlative trends and mathematical expressions of the measured porosity on the permeability coefficient, compressive strength at 7-days, compressive strength at 28-days, and splitting tensile strength were analyzed. The coefficient of determination (R^2^) assessed goodness-of-fit for these empirical relationships, providing insights into the trends for future pervious concrete mix design and analysis.

First, the fit between the measured porosity and the permeability coefficient (PC) is shown in Figure 13a. An exponential growth function y = 0.7122e^0.105*x*^ was fitted, achieving an excellent R^2^ of 0.9148. This function effectively captures the non-linear correlation between permeability and porosity. The underlying physics relates to percolation theory: increasing porosity significantly enhances the proportion and connectivity of internal pores, forming more complete flow networks, leading to an exponential rise in permeability. Beyond a critical porosity, even small increases can cause large permeability jumps, characteristic of the transition from “non-permeable” to “fully permeable” states in porous media. This empirical relationship can help understand the trend of permeability change with porosity. Figure 13b shows the fit for measured porosity and 7 d CS using an exponential decay model: y = 70.195e^−0.068*x*^ (R^2^ = 0.678, moderate fit). Unlike permeability, strength decreases with increasing porosity due to reduced compactness, effective load-bearing area, and paste coating efficiency, hindering hydration and promoting microcracking. The exponential form suggests that the detrimental effect is most sensitive at lower porosities, stabilizing later, highlighting the inherent conflict between permeability and strength. Figure 13c shows a power law function for 28 d CS: y = 396.46x^−0.905^ (R^2^ = 0.5465). Although R^2^ is lower than that for 7 d, the model has explanatory power. The power law indicates a progressively diminishing effect of porosity on strength loss at higher porosities, possibly due to partial pore filling by later hydration products. The lower R^2^ suggests that other factors (e.g., SR, w/c ratio) also significantly influence 28 d CS, indicating that this relationship is primarily descriptive for the tested data. Figure 13d shows a logarithmic decay function for STS: y = −1.848 ln(x) + 7.8215 (R^2^ = 0.549). This form reflects a “rapid decline—slow stabilization” response to porosity, consistent with crack propagation: initial pore increases create many stress concentrators, rapidly reducing tensile strength; at higher porosities, crack paths stabilize, slowing strength loss. The logarithmic function indicates that sensitivity is concentrated in the medium-low porosity range (14%~20%), which should be carefully managed for structural safety. These fitted relationships are derived from the current dataset and serve to quantify the observed correlations rather than to predict performance beyond the tested conditions.

These relationships quantify observed correlations within the tested range and should not be confused with generalized predictive models, which require independent validation on broader datasets.

### 3.4. Research Novelty

This study provides several unique contributions that distinguish it from previous works on pervious concrete. Firstly, it integrates both single-factor experiments and an L_9_(3^3^) orthogonal design framework to systematically evaluate the influence of porosity, water–cement ratio, sand rate, and viscosity-modifying admixture (VMA) dosage on mechanical and hydraulic performance. Secondly, the research highlights the coupled role of fine sand particles in modifying pore structure—occupying void spaces rather than directly clogging pores—thereby clarifying their dual effect on permeability and strength. Thirdly, the study combines empirical correlation fitting with Spearman correlation analysis to quantitatively describe the relationships between porosity, permeability, and strength, offering insights into mechanistic trends. Fourthly, the identification of an optimized mix proportion, achieving both high compressive strength (43.5 MPa) and adequate permeability (2.57 mm·s^−1^), demonstrates a practical pathway for developing high-strength pervious concrete. Finally, the methodological integration of experimental design and statistical analysis establishes a transferable framework for guiding the sustainable development of porous cement-based materials, with potential applications in urban infrastructure and ecological construction.

## 4. Conclusions

This study systematically investigated the effects of target porosity, water–cement (w/c) ratio, sand rate, and viscosity-modifying admixture (VMA) dosage on the permeability and mechanical performance of pervious concrete through single-factor tests and orthogonal experimental design. The key conclusions are as follows:

(1) Target porosity is the dominant factor governing both permeability and strength. Higher porosity markedly enhances permeability but significantly reduces compressive and tensile strengths. This fundamental trade-off defines the performance boundaries of pervious concrete.

(2) The water–cement ratio exhibits a dual effect: permeability peaked at w/c = 0.30, whereas maximum compressive strength occurred at w/c = 0.32. This indicates that a narrow optimal range exists to balance hydraulic and mechanical performance.

(3) Sand incorporation improves strength but reduces permeability. A moderate sand rate (≈6–7%) strengthens the interfacial transition zone and aggregate packing, yielding improved compressive and tensile properties while maintaining acceptable permeability. Excessive sand content, however, drastically reduces hydraulic capacity.

(4) VMA dosage plays a minor but stabilizing role. At 0.01%, VMA improved workability and bonding, optimizing pore structure and mechanical properties without significantly altering permeability.

(5) Orthogonal testing identified an optimal mix (w/c = 0.30, sand rate = 7%, VMA = 0.01%, target porosity ≈ 15%), which achieved a compressive strength at 28-days of 43.5 MPa and permeability coefficient of 2.57 mm·s^−1^, demonstrating the feasibility of producing high-strength pervious concrete with adequate permeability.

(6) Correlation analysis revealed strong empirical relationships between the measured porosity and material performance. Permeability exhibited exponential growth with porosity, while compressive and tensile strengths showed negative exponential, power-law, or logarithmic trends. These findings provide mechanistic insights but are not intended as generalized predictive models.

**Advances to the state of the art:**

This work contributes a systematic framework that integrates single-factor evaluation, orthogonal design, and correlation analysis for pervious concrete optimization. Unlike many previous studies focusing solely on permeability-strength trade-offs, this research quantitatively demonstrated how design porosity and fine aggregate incorporation interact with the w/c ratio and VMA to achieve balanced performance. The combination of orthogonal experiments with Spearman correlation analysis offers a novel methodological approach for guiding mix proportioning in sustainable infrastructure applications.

**Future research directions:**

Future studies should extend this framework to explore (i) the use of recycled aggregates and industrial by-products for low-carbon pervious concrete, (ii) durability performance under freeze–thaw, clogging, and long-term service conditions, and (iii) large-scale field validation to bridge the gap between laboratory optimization and engineering practice.

## Figures and Tables

**Table 1:** Key characteristics and challenges of pervious concrete.

Aspect.	Description	Influencing Factors	Common Challenges
Porosity	Typically 15–30%	Aggregate gradation, compaction, paste volume	Trade-off with strength
Permeability	Governed by pore connectivity	Porosity, pore size distribution, clogging	Sensitivity to compaction and segregation
Strength	Lower than conventional concrete	Paste quality, aggregate interlock, ITZ density	Weak interfacial transition zone (ITZ)
Mix Design	Low fine aggregate, high paste coating	w/c ratio, admixtures, sand content	Difficulty in achieving uniform paste distribution

**Table 2:** Mix proportions for single-factor experiments.

Series	No.	Target Porosity (%)	Cement Kg/m^3^	Aggregate kg/m^3^	W/C Ratio	PCE (%)	VMA (%)	Sand Rate (%)
TP	TP1	14	463	1627	0.3	0.12	0.01	–
	TP2	17	430					–
	TP3	20	399					–
	TP4	23	366					–
	TP5	26	334					–
WC	WC1	20	480	1627	0.26	0.12	0.01	–
	WC2	20	480	1627	0.28	0.12	0.01	–
	WC3	20	480	1627	0.30	0.12	0.01	–
	WC4	20	480	1627	0.32	0.12	0.01	–
	WC5	20	480	1627	0.34	0.12	0.01	–
SR	SR0	20	480	1627	0.30	0.12	0.01	0
	SR1	20	480	1594	0.30	0.12	0.01	2
	SR2	20	480	1562	0.30	0.12	0.01	4
	SR3	20	480	1529	0.30	0.12	0.01	6
	SR4	20	480	1497	0.30	0.12	0.01	8
	SR5	20	480	1464	0.30	0.12	0.01	10
VMA	VMA1	20	480	1627	0.30	0.12	0	–
	VMA2	20	480	1627	0.30	0.12	0.005	–
	VMA3	20	480	1627	0.30	0.12	0.01	–
	VMA4	20	480	1627	0.30	0.12	0.015	–
	VMA5	20	480	1627	0.30	0.12	0.02	–

Note: TP = Target Porosity series; WC = Water–Cement Ratio series; SR = Sand Rate series; VMA = Viscosity-Modifying Admixture series; PCE = Polycarboxylate superplasticizer.

**Table 3:** Factor level table of the orthogonal experimental design.

Level	Factor A	Factor B	Factor C
	Sand Rate (%)	VMA (%)	Target Porosity (%)
1	4	0.015	14
2	6	0.01	20
3	8	0.005	26

**Table 4:** Orthogonal test layout L_9_(3^3^).

No.	Factor A	Factor B	Factor C
	Sand Rate (%)	VMA (%)	Target Porosity (%)
OED1	4	0.015	14
OED2	4	0.01	20
OED3	4	0.005	26
OED4	6	0.01	26
OED5	6	0.005	14
OED6	6	0.015	20
OED7	8	0.005	20
OED8	8	0.015	26
OED9	8	0.01	14

**Table 5:** Orthogonal test results.

No.	7 d /MPa	28 d /MPa	Splitting Tensile Strength /MPa	Permeability Coefficient /mm·s^−1^
OED1	22.9	31.3	2.35	4.6
OED2	19.3	30.3	2.67	5.5
OED3	16.5	23.1	2.46	9.1
OED4	9.5	16.8	2.12	8.7
OED5	27.4	36.1	2.78	4.1
OED6	14.6	20.8	2.32	5.2
OED7	24.3	33.3	2.35	4.9
OED8	18.6	31.3	2.62	8.5
OED9	33.2	42.6	3.33	2.4

**Table 6:** Range analysis of the permeability coefficient by orthogonal test.

No.	Factors				Permeability Coefficient /mm·s^−1^
	A: Sand Rate (%)	B: VMA (%)	C: Target Porosity (%)	Null Column	
OED1	1(4)	1(0.015)	1(14)	1	4.6
OED2	1	2(0.01)	2(20)	2	5.5
OED3	1	3(0.005)	3(26)	3	9.1
OED4	2(6)	2	3	1	8.7
OED5	2	3	1	2	4.1
OED6	2	1	2	3	5.2
OED7	3(8)	3	2	1	4.9
OED8	3	1	3	2	8.5
OED9	3	2	1	3	2.4
K1	6.40	6.10	3.70	6.07	
K2	6.00	5.53	5.20	6.03	
K3	5.27	6.03	8.77	5.57	
R	1.13	0.57	5.07	0.50	

**Table 7:** Analysis of variance (ANOVA) for the permeability coefficient.

Factors	Deviation Square Sum	Degrees of Freedom	F Ratio	Significance Level
Sand rate	1.98	2	4.23	Non-significant
VMA	0.58	2	1.23	Non-significant
Target porosity	40.64	2	86.66	Significant
Error	0.47	2		

**Table 8:** Range analysis of mechanical properties by orthogonal test.

No.	Factors				CS of 7 d (MPa)	CS of 28 d (MPa)	STS (MPa)
	Sand Rate (%)	VMA (%)	Target Porosity (%)	Null Column			
OED1	1 (4)	1 (0.015)	1 (14)	1	22.9	31.3	2.35
OED2	1	2 (0.01)	2 (20)	2	19.3	30.3	2.67
OED3	1	3 (0.005)	3 (26)	3	16.5	23.1	2.46
OED4	2 (6)	2	3	1	9.5	16.8	2.12
OED5	2	3	1	2	27.4	36.1	2.78
OED6	2	1	2	3	14.6	20.8	2.32
OED7	3 (8)	3	2	1	24.3	33.3	2.35
OED8	3	1	3	2	18.6	31.3	2.62
OED9	3	2	1	3	33.2	42.6	3.33
K1 (7 d)	19.55	18.67	27.80	18.91			
K2 (7 d)	17.16	20.64	19.38	21.74			
K3 (7 d)	25.36	22.76	14.89	21.41			
R (7 d)	8.20	4.09	12.91	2.83			
K1 (28 d)	28.23	27.80	36.67	27.13			
K2 (28 d)	24.57	29.90	28.13	32.57			
K3 (28 d)	35.73	30.80	23.73	28.83			
R (28 d)	11.17	3.03	12.93	5.43			
K1 (STS)	2.49	2.43	2.82	2.28			
K2 (STS)	2.41	2.71	2.45	2.69			
K3 (STS)	2.77	2.53	2.40	2.70			
R (STS)	0.36	0.28	0.42	0.43			

**Table 9:** ANOVA for compressive strength at 7-days.

Factors	Deviation Square Sum	Degrees of Freedom	F Ratio	Significance Level
Sand rate	106.78	2	7.43	Non-significant
VMA	25.04	2	1.74	Non-significant
Target porosity	257.83	2	17.93	Significant
Error	14.38	2		

**Table 10:** ANOVA for compressive strength at 28-days.

Factors	Deviation Square Sum	Degrees of Freedom	F Ratio	Significance Level
Sand rate	194.30	2	13.42	Significant
VMA	15.40	2	1.00	Non-significant
Target porosity	257.39	2	17.91	Significant
Error	15.40	2		

**Table 11:** ANOVA for 28-day splitting tensile strength.

Factors	Deviation Square Sum	Degrees of Freedom	F Ratio	Significance Level
Sand rate	0.36	2	89.75	Significant
VMA	0.11	2	28.50	Significant
Target porosity	1.21	2	303.25	Significant
Error	0	2		

**Table 12:** Mix proportions for the significance optimization test.

No.	Target Porosity (%)	Cement Kg/m^3^	Aggregate kg/m^3^	W/C Ratio	PCE (%)	VMA (%)	Sand Rate (%)
YZ0	15	480	1497	0.3	0.12	0.01	8
YZ1			1513				7
YZ2			1481				9

**Table 13:** Results of the significance optimization test.

No.	7 d /MPa	28 d /MPa	Splitting Tensile Strength /MPa	Permeability Coefficient /mm·s^−1^
YZ0	35.0	42.9	3.49	2.43
YZ1	37.8	43.5	3.51	2.57
YZ2	33.0	41.8	3.3	2.17

**Figure A1:**
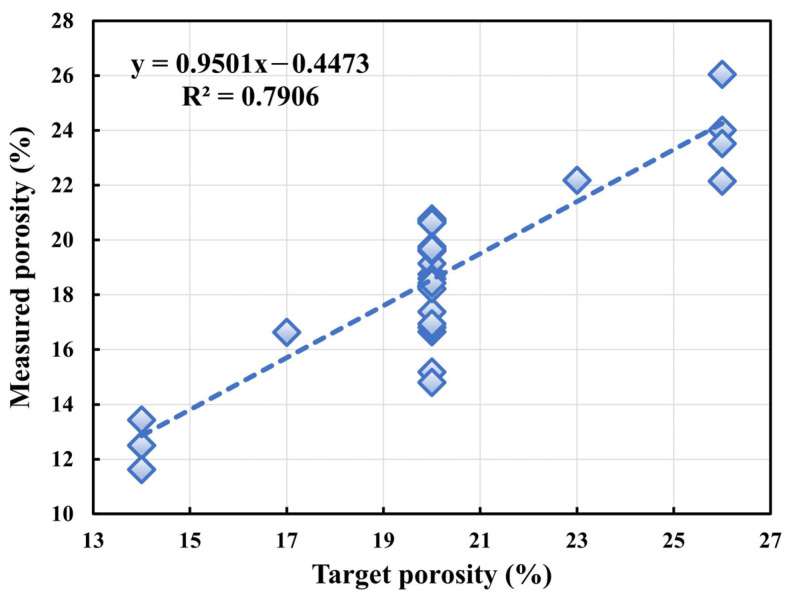
Correlation analysis between target porosity and measured porosity.

## Data Availability

The original contributions presented in this study are included in the article. Further inquiries can be directed to the corresponding author.

## Appendix A. Analysis of Target and Measured Porosity

### Appendix A.1. Design Methodology for Target Porosity

The target porosity (*P_T_*) in this study was designed using the volumetric method, which is a fundamental and widely adopted approach for pervious concrete mix proportioning. The design is based on the principle that the total volume of a concrete mixture is the sum of the absolute volumes of its solid constituents (cement, aggregate, supplementary cementitious materials) and the designed entrained air voids (i.e., the target pores).

The target porosity was calculated using the following equation:

$$P_{T}=\left[1-\left(V_{agg}+V_{cem}+V_{water}+V_{other}\right)\right]\times 100\%$$

where *P_T_* is the target porosity (%). *V_agg_* is the absolute volume of coarse and fine aggregate (m^3^). *V_cem_* is the absolute volume of cement (m^3^). *V_water_* is the absolute volume of mixing water (m^3^). *V_other_* is the absolute volume of other solid constituents (e.g., VMA, superplasticizer).

The absolute volume of each component was calculated from its mass and specific gravity. By fixing the total volume of the mixture (e.g., 1 m^3^) and the target porosity value, the required mass of aggregates and paste could be determined. This method allows for precise theoretical control over the intended void structure of the concrete before mixing.

### Appendix A.2. Correlation and Discrepancy Between Target and Measured Porosity

The measured porosity (*P_M_*) was determined experimentally on hardened specimens using the gravimetric method according to standard protocols (e.g., ASTM C1754), which involves measuring the bulk density of the saturated-surface-dry specimen and its dry density to calculate the total volume of permeable void space.

The relationship between the designed target porosity (*P_T_*, independent variable) and the experimentally measured porosity (*P_M_*, dependent variable) was analyzed through linear regression, as shown in Figure A1.

The analysis yielded the following fitting equation: *P_M_* = 0.9501 *P_T_*—0.4473 (R^2^ = 0.7906). This equation reveals a strong linear correlation between the designed and measured values, as evidenced by the high coefficient of determination (R^2^ = 0.7906). The slope of the regression line (0.9501) is very close to 1, indicating that the measured porosity increases at nearly the same rate as the target porosity. This strong correlation validates the effectiveness of the volumetric method in reliably designing pervious concrete mixes with a predictable pore structure.

However, the regression equation also reveals a consistent, systematic discrepancy: for any given target porosity, the measured porosity is predictably slightly lower. The sources of this consistent difference are multifaceted and inherent to the transition from theoretical design to practical reality:

(1) Closed and Semi-Connected Pores: The target porosity (*P_T_*) represents the total designed void space. In contrast, the standard gravimetric measurement method (*P_M_*) primarily captures the interconnected pores accessible to water. A population of closed pores (entrapped air within the paste) and semi-connected pores are created during mixing but are not filled with water during saturation. These pores contribute to *P_T_* but are not included in *P_M_*.

(2) Paste Drainage and Compaction Effects: During the placing and compaction phases, some degree of paste drainage can occur. The cement paste may settle downward, slightly reducing the void volume in the upper sections of the specimen. Furthermore, the standardized compaction effort can marginally reduce the overall pore volume compared to the ideal, uncompacted state assumed in the theoretical design.

(3) Pore Blocking by Fines: The movement of very fine particles (from cement, VMA, or aggregate fines) during mixing and vibration can lead to the partial blocking of some pore throats. This can make certain interconnected pores inaccessible to water during the saturation phase, effectively causing them to be excluded from the *P_M_* measurement.
